# Pathways to a Shiny Future: Building the Foundation for Computational Physical Chemistry and Biophysics in 2050

**DOI:** 10.1021/acsphyschemau.4c00003

**Published:** 2024-04-04

**Authors:** Denys Biriukov, Robert Vácha

**Affiliations:** † CEITEC − Central European Institute of Technology, Masaryk University, Kamenice 753/5, 625 00 Brno, Czech Republic; ‡ National Centre for Biomolecular Research, Faculty of Science, Masaryk University, Kamenice 753/5, 625 00 Brno, Czech Republic; ¶ Department of Condensed Matter Physics, Faculty of Science, Masaryk University, Kotlářská 267/2, 611 37 Brno, Czech Republic

**Keywords:** molecular dynamics, multiscale modeling, artificial intelligence, simulation databases, computational physical chemistry, computational biophysics

## Abstract

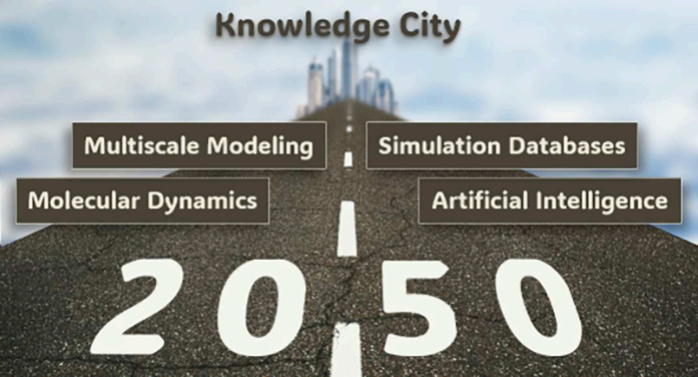

In the last quarter-century, the field of molecular dynamics (MD) has undergone a remarkable transformation, propelled by substantial enhancements in software, hardware, and underlying methodologies. In this Perspective, we contemplate the future trajectory of MD simulations and their possible look at the year 2050. We spotlight the pivotal role of artificial intelligence (AI) in shaping the future of MD and the broader field of computational physical chemistry. We outline critical strategies and initiatives that are essential for the seamless integration of such technologies. Our discussion delves into topics like multiscale modeling, adept management of ever-increasing data deluge, the establishment of centralized simulation databases, and the autonomous refinement, cross-validation, and self-expansion of these repositories. The successful implementation of these advancements requires scientific transparency, a cautiously optimistic approach to interpreting AI-driven simulations and their analysis, and a mindset that prioritizes knowledge-motivated research alongside AI-enhanced big data exploration. While history reminds us that the trajectory of technological progress can be unpredictable, this Perspective offers guidance on preparedness and proactive measures, aiming to steer future advancements in the most beneficial and successful direction.

## Introduction

In just 26 years, we will reach the year 2050. Reflecting back to 1998, 26 years ago, the field of molecular dynamics has dramatically transformed from its pioneering days of simulating mainly hundreds of atoms over several nanoseconds.^1,2^ Currently, we operate with intricate and multiscale models involving millions of atoms/particles simulated over the microsecond time scale.^3^ Such remarkable progress, marking a significant leap in computational physical chemistry, has been fueled by substantial technological, hardware, and methodological advancements. This evolution is poised to continue and even accelerate with the integration of artificial intelligence (AI) concepts, optimizing human efforts in the preparation, execution, analysis, and even review of MD simulations.

**Figure 1:**
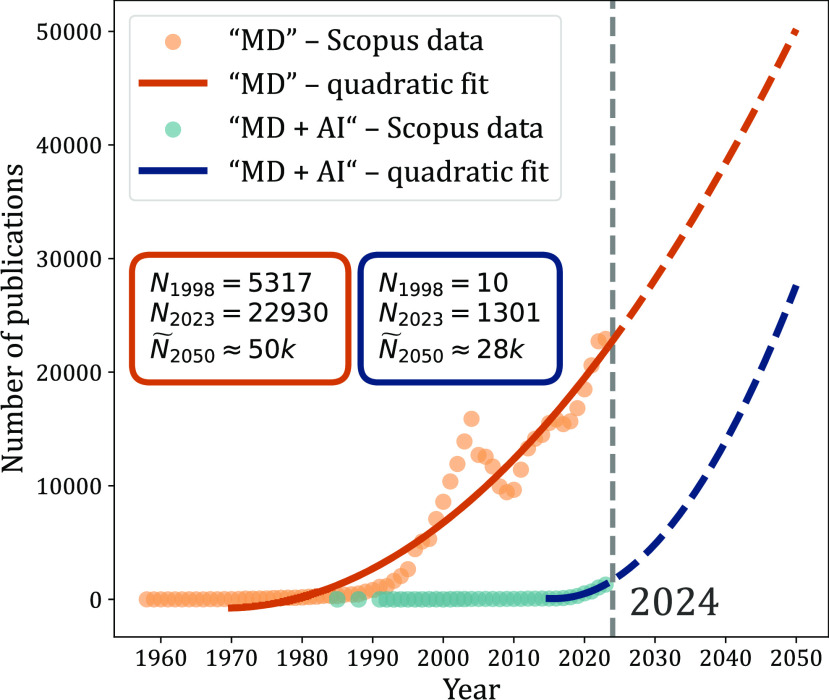
Molecular dynamics simulations and artificial intelligence in scientific publishing. Data collected from Scopus as of December 21, 2023. The search was based on finding occurrences of “molecular dynamics” either alone (“MD”) or in combination with “artificial intelligence”, “machine learning”, “neural network”, and “deep learning” phrases (“MD + AI”) within the title, abstract, or keywords of publications indexed in the Scopus database. The collected data were fitted and extrapolated to the year 2050. The projected values, , are shown in the inlet rectangles and compared with publication data from the years 1998 and 2023 (*N*_1998_ and *N*_2023_, respectively). The utilized Scopus data set is available on Zenodo under DOI: 10.5281/zenodo.10673694.

However, this progression has its challenges. The number of simulation studies and, thereby, the volume of data generated by the expanding scientific community are constantly growing, Figure 1, risking the possibility of valuable insights being lost in an overwhelming amount of information. The complexity of models is also increasing, heightening the risk of “garbage in–garbage out” scenarios due to potential mistakes in the preparation phase. With the continuously growing impact of MD simulations in, for instance, biomedical applications,^4,5^ the potentially incorrect or misleading results can have increasingly severe consequences. Therefore, a key challenge for computational physical chemists (and also scientific publishers) by 2050 will be effectively utilizing, managing, and critically reviewing this wealth of data.^6^

The role of AI in shaping the future of many fields, including computational physical chemistry, also becomes apparent. AI is already often coupled to MD simulations: the noticeable growth of combined MD and AI studies started less than ten years ago, and further AI’s involvement is likely to surge dramatically, Figure 1. While this combination of methods is promising to expedite groundbreaking discoveries, the integration of AI is set to further amplify the volume of data generated, potentially reaching scales unmanageable by human researchers alone. This scenario suggests a future in which AI-driven simulations become so advanced and numerous that their review and analysis may be feasible only with the assistance of, or entirely by, equally sophisticated AI systems. Moreover, such AI-driven analysis could become essential in formulating initial hypotheses and recommending simpler simulations to verify or refute them.

In this Perspective, we envision a future in 2050 (elaborating on current trends and necessary actions to take) where every MD simulation can be traced through global open-access databases integrated with sophisticated AI models. These databases, ideally encompassing every conceivable MD simulation, would link to peer-reviewed publications or open-access platforms housing detailed simulation data and additional necessary information. They would also accommodate nonpublished, failure, and even “wrong” data, thus reducing redundant efforts and encouraging a more comprehensive scientific dialogue. Advanced AI models in this envisioned future would not only organize these data but also evaluate, refine, and improve simulation force fields by aligning them with experimental data and cross-validating various model resolutions, such as all-atom vs coarse-grained simulations. Furthermore, these models would autonomously conduct new MD simulations, identify inconsistencies, suggest enhanced parameters or even new phenomena, and enable the modeling of increasingly complex and multiscale systems. However, realizing this vision requires a monumental collaborative effort involving back-tracking—or rather regenerating according to Moore’s law—likely millions of existing simulation trajectories and fostering open scientific collaboration worldwide—a necessary “entrance fee to 2050” for securing a bright future in computational physical chemistry.

For conciseness and focus, this Perspective primarily discusses molecular simulations within biological systems that bridge the fields of physical chemistry and biophysics. Biological systems, with their diverse sizes and time scales, provide a rich context for discussing computational advancements. For instance, compare the first ever one-microsecond-long simulation capturing the folding of a 36-residue-long peptide in 1998^7^ and the 500 ps long simulations of a 64-dipalmitoylphosphatidylcholine bilayer a year earlier^8^ with today’s simulations where a 1 μs duration is often the minimum benchmark for force field comparison.^9^ Moreover, modern simulations frequently can involve much larger proteins embedded in multicomponent lipid bilayers containing hundreds of lipids.^10,11^ Current biological simulations set even more ambitious milestones, both in terms of simulation length (ranging from microseconds to seconds) and system size and complexity—from realistic cellular membranes^12−15^ to an entire ribosome,^16^ minimal cell,^17^ virus capsid,^18,19^ or envelope structure of SARS-CoV-2.^20^ These and many other examples provide a valuable lens through which to view the evolution and future of MD simulations.

This Perspective does not delve into the technical details or listing available AI models, and we refer instead to a large number of recent works, overviews, and special issues exclusively dedicated to this topic, see, e.g., refs (21−33) and references therein. We rather focus on the practical applications of AI concepts, presuming that by 2050, AI models will achieve unprecedented levels of sophistication, and we need just to choose which ones to use or allow AI to make these decisions for us. We briefly discuss the current state of molecular dynamics simulations, the emerging challenges in data storage, the potential application of AI, and what steps should be undertaken by 2050 or even right now. For clarity, in this Perspective, the term “AI” typically refers to a variety of terms, including “machine learning”, “neural networks”, “large language models”, and others. Though not technically precise, this simplifies our discussion without compromising the overall understanding.

It is also important to recognize the uncertainties inherent in forecasting over such an extended time frame. As we stand in 2024, our vision for 2050 is shaped by the developments we observe today. The unpredictable nature of technological evolution, especially in a field as dynamic as AI, means that the future may significantly deviate from our current expectations. Nevertheless, certain trends and necessary actions are already evident, and here, we aim to underscore the importance of adaptability in navigating the constantly evolving scientific landscape.

## Current Prospects and the Need of Artificial Intelligence

We live in an era marked by the continuous and likely exponential growth of information including scientific data. Individual scientists or even large research teams cannot feasibly process it all. Even now, it is close to, if not impossible, for a scientist to read or browse through all of the new relevant articles in the field of interest, let alone examine the publicly available MD simulations. Therefore, developing tools that enable researchers to stay up to date becomes a practical necessity.

**Figure 2:**
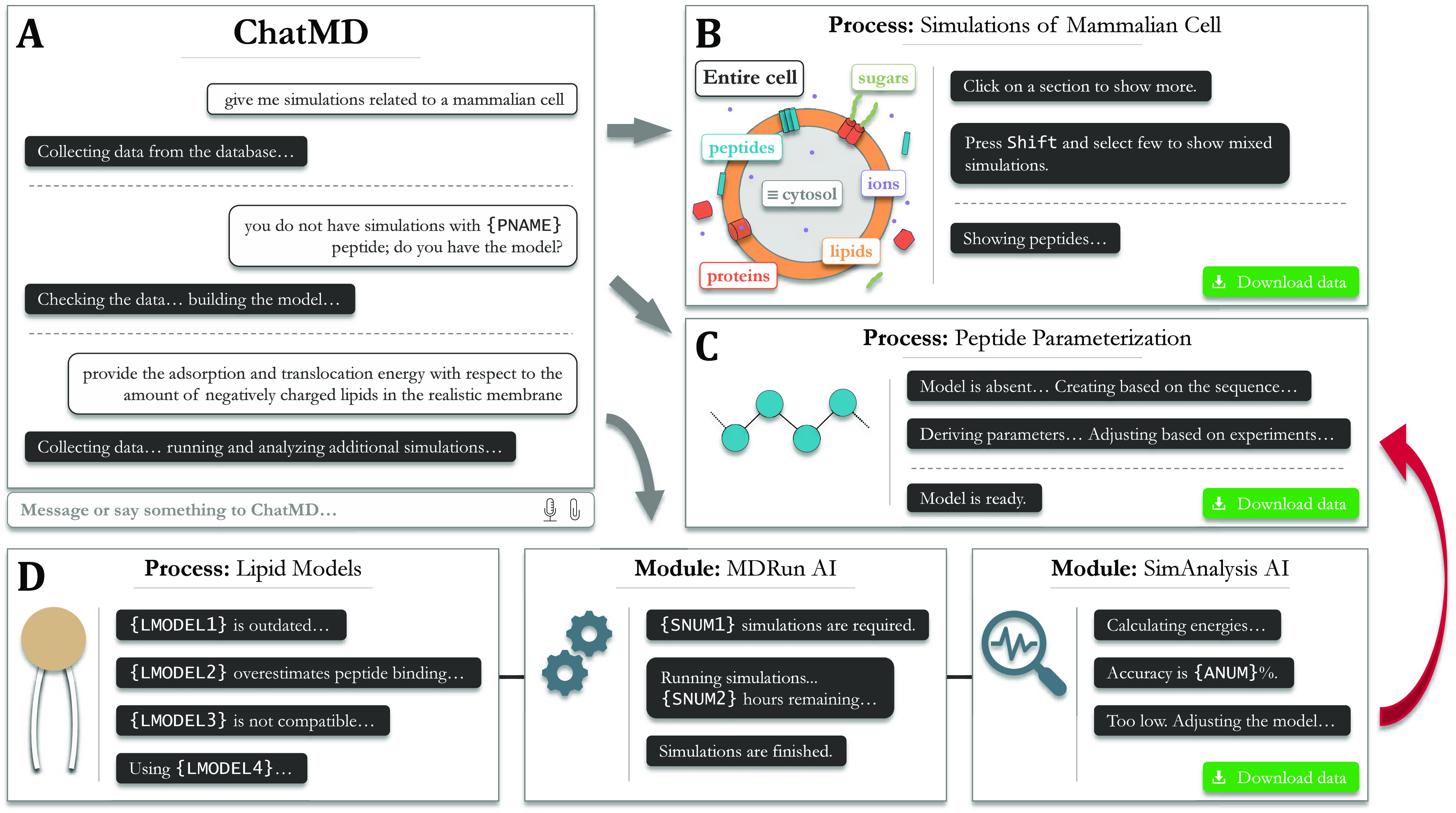
Possible schematic interface of a futuristic molecular dynamics AI-maintained engine capable of setting up, running, and analyzing simulations. (A) Initial user-interaction interface. (B) Dashboard of molecular dynamics simulations of mammalian cells. (C) New molecule parametrization interface: adding an unlisted peptide. (D) Interface of a membrane model selection compatible with a new peptide model followed by simulation and analysis interfaces.

While preferences for such tools are subjective, the successful existing tools for similar purposes are typically intuitive, straightforward to use, and visually appealing. Most likely, precisely these characteristics contributed to the widespread popularity of AI-managed user-chat interfaces like ChatGPT (https://chat.openai.com), Bard with its most recent Gemini AI model (https://bard.google.com/), or Copilot (https://copilot.microsoft.com/). Adopting and advancing a similar idea for specific scientific purposes seems to be an optimal solution for the near future. The chat-like approach could revolutionize the initial interaction with scientific information or even performing MD simulations, see Figure 2 for a possible future example, making AI the primary tool for administration and search functions. The final data may still be presented in traditional formats, such as tables or diagrams, to meet conventional expectations for scientific data display.

While the prospect of AI models managing MD simulations in the coming years is feasible, verifying their consistency and accuracy is a more profound challenge. The adage “garbage in, garbage out” is particularly pertinent. It is crucial to acknowledge that simulation results are affected not just by the chosen model (called “force field” in classical MD) but also by subtle variations in simulation protocols, software choices, and data analysis methods.^34,35^ Minor discrepancies might be inconsequential on a smaller scale, but an AI model trained on extensive data sets could amplify these errors. This could result in “hallucinating simulations or force fields” that appear to generate at first glance realistic but ultimately inaccurate results.

A less acknowledged issue is whether the converse concept—good in, good out—always holds true. The answer is not necessarily affirmative since a successful combination of a model and simulation protocol for one system does not guarantee success for even a slightly altered system.^36,37^ An intriguing consideration, for example, is whether current atomistic force fields can maintain the stability of secondary and tertiary protein structures over millisecond time scales.^38^ It is possible that some, or perhaps all, of the force fields may not be adequate for the next era of simulations. Similar concerns apply to MD simulation codes: extended time scales of larger systems are already revealing numerical inaccuracies and bugs that are usually not apparent in smaller systems and shorter simulations.^39^

Overall, the key is to carefully select a model that is appropriate for the specific application.^40^ Currently, most large-scale simulations rely on established models extensively validated by the scientific community. These simulations yield groundbreaking insights despite the inherent model limitations; even all-atom force fields are essentially a coarse-grained representation of quantum reality. Typically, the model limitations are identified, and potential solutions are proposed.^41−43^ However, these solutions could be system-dependent or even not widely known and not incorporated into the standard protocols associated with the original model. Such circumstances lead to a complex sequence of updates and inconsistencies between simulations that can be difficult to track. This problem can be mitigated by implementing open-science practices aimed to collect simulations into dedicated repositories for cross-evaluation^44−46^ (see section Challenges in Data Management for representative examples such as NMRlipids^46^). In all these scenarios, AI can play an invaluable role in model comparison and validation, offering insights and guidance that might otherwise be unattainable even when performing replica simulations to prevent “false positive” conclusions.^47^

Beyond MD models and simulations *per se*, related tools like AlphaFold^48^ are also expected to evolve and advance further. Apart from known strengths and limitations of the current AlphaFold2,^49−51^ the main fundamental concern is that the single structure does not represent functional motions of a protein.^52^ Therefore, future tools might be capable of predicting the dynamics of molecules, offering more than a static snapshot of their structures (one might suggest a title like “AlphaDynamics”). The involvement of MD concepts in such tools is very promising, and the first steps have already been made, see, e.g., ref (53), where multiple Boltzmann-ranked ensembles from a single protein sequence can be generated and then propagated by MD. However, the quality of these tools will again rely on the quality of reference data that might be more ambiguous due to the different models and resolutions used and time scales explored.

This leads us to envision global AI-administrated databases as self-expanding and self-correcting entities, not merely repositories awaiting human input. Integrated AI within these databases should identify errors and inconsistencies. AI could also try to autonomously fix them by performing missing simulations or asking for human intervention to distinguish possible errors from yet-unknown phenomena. Without AI functionalities, the substantial efforts invested in creating these databases may not be fully exploited, especially given the continuous refinement of the models and the generation of new simulations. In the end, we could be equipped with AI tools that organize data, allow “communication” with them, verify and double-check accuracy, and fill in missing gaps without explicit requests. The human role would then be as a moderator who applies sanity checks, makes major decisions and critical adjustments, and distills the key insights, essentially trying to make sense of AI-proposed data interpretations. In modern terms, a scientist should be able to effectively “prompt” the AI to provide or generate information or simulation and interact with it until a desirable and meaningful outcome is achieved. Realizing this vision requires thoughtful and careful implementation of technologies to ensure widespread adoption and success.

## Coupling Molecular Dynamics and Artificial Intelligence

To grasp how AI can enhance MD simulations, it is helpful to characterize the current state and typical applications. Below, we discuss MD simulations along several critical points, which, looking ahead to 2050, are anticipated to evolve dramatically with the help of AI.

First of all, the model resolution is a crucial factor determining the underlying accuracy and accessible scales of MD simulations.^54^ It can be categorized into the following: (i) *ab initio* molecular dynamics, where the nuclei are treated as classical particles, while the electronic structure and interatomic forces are calculated from first-principles;^55^ (ii) all-atom molecular dynamics, where each atom (or a ghost/virtual particle) is modeled as a sphere with a constant partial charge, while interatomic interactions are derived from (semi)empirical potentials; (iii) coarse-grained molecular dynamics, where multiple atoms are united into larger units called “beads” and further used to construct small biological entities such as amino acids, nucleotides, or saccharides (see, e.g., popular Martini,^56^ SPICA,^57^ or SIRAH^58^ force fields); interactions in coarse-grained models are often described using potentials similar to those in all-atom simulations; (iv) ultra coarse-grained models with implicit solvent, representing large biological entities with arbitrary building blocks,^59,60^ while interactions, including their form, are specifically tailored for particular purposes or systems of interest.

Selecting the appropriate model resolution is typically the first step in the design of a research plan. This choice determines the simulation’s capabilities, namely, which system’s properties can be accurately described, if at all. Each of the listed approaches has significantly contributed to the development of MD simulations, as illustrated by various examples spanning from 1998 until now; see Table 1. These examples essentially represent “(among) first-of-their-kind” events, potentially viewed as significant milestones within the field, in terms of scientific discoveries or methodological innovations.

**Table 1:** Examples of Notable Molecular Dynamics Studies

method	system and simulated time	landmark	reference
**1998**			
Car–Parrinello *ab initio* MD	glucose in 58 waters, several ps	first *ab initio* MD simulation of a solvated glucose	(61)
all-atom force field MD	HP-36 peptide in ∼3000 waters, 1 μs	first μs-long simulation of a protein folding	(7)
all-atom force field MD	N/A	presentation of CHARMM22 all-atom force field for proteins	(62)
coarse-grained MD	a few hundreds CG particles, up to 5 μs^a^	pioneering CG simulation of a lipid bilayer self-assembly	(63)
			
**2022**–**2023**			
Born–Oppenheimer *ab initio* MD	chignolin in water, 10,000 × 225 fs	fully explored conformational space of a 10-residue protein at *ab initio* MD level	(64)
all-atom force field MD	N/A	description of Anton 3 supercomputer enabling ms-long biosimulations	(65)
all-atom MD	∼44 million atoms, several ns	atomistic simulation of HIV capsid using machine-learning potential	(66)
coarse-grained MD	∼37 million CG particles, 1 μs	a coarse-grained simulation of the entire SARS-CoV-2 envelope	(20)
coarse-grained MD	∼550 million CG particles, not simulated	first coarse-grained Martini model of an entire cell	(17)
			
**2050**			
**Multiscale ms-long simulations of an entire cell?**			

a Approximate time scale.

While the progress over the last years is evident, simulations incorporating multiple resolutions within a single system generally advance more slowly due to their methodological and technical complexity. In the future, we may witness the emergence of advanced hybrid methodologies like AI-accelerated multiscale simulations. These simulations might adopt varying resolution levels for different system parts (for example, a quantum description of active sites while the remainder is described classically), dynamically adjust them, and even attempt to extrapolate system behavior to extend the time scale. In current terms, this possible approach could be termed as the automatically generated and regulated on-the-fly version of quantum mechanics/molecular mechanics, or simply the future iteration of it.^67,68^ Such simulations would potentially facilitate the modeling of processes, where both the quantum description and associated conformational changes are crucial, e.g., enzyme catalysis^69^ or electron transfer in proteins.^70^ Similarly, coupling AA and CG resolutions in a single simulation would improve the accessible time scales while maintaining atomistic accuracy where necessary. Although AA/CG models already exist,^71−73^ they again lack the capability for on-the-fly adjustment of resolution partitioning. However, this challenge is primarily technical and appears to be surmountable with the integration of AI algorithms.

The fusion of simulations with experiments is also expected to become more seamless, with AI playing a pivotal role in bridging these two realms such as suggesting new simulations or experiments for improved correlation. The cornerstone of molecular simulations is gaining the information not accessible by the available experimental methods, e.g., the simulation is actually a stand-alone research aimed at elucidating mechanisms of various phenomena or making predictive analyses. However, simulations can also play a synergistic role in conjunction with experimental studies, i.e., the information gathered by experiments could focus simulations and *vice versa*.^74^ For instance, MD simulations can be performed imposing restraints from NMR or cryogenic electron microscopy experiments.^75,76^ To enhance that further, we anticipate a broader integration of high-performance computing and AI, fostering a more complex interplay. This integration may be particularly fascinating as simulations and experiments typically drift toward each other: simulations strive to characterize larger biological assemblies over extended time scales, whereas the most recent experimental techniques increase the resolution to capture events occurring over shorter periods. Together, these approaches should aim to replicate *in vivo*, i.e., biologically relevant systems and conditions as closely as possible.

The model and method development is also a vital part of computational physical chemistry, which can greatly benefit from AI concepts. The (semi)automatized or/and AI-driven model/force field generation is already under extensive development^77−91^ but yet to be tested beyond the current “comfort zone” of a limited range of systems. While methodological studies frequently incorporate experimental data, more often than not, they repurpose already available data from well-understood systems. A classic example is the alanine dipeptide—a small, thoroughly studied system where a biomolecule exhibits rare events in solution at room temperature. It is typically used for benchmarking various enhanced sampling methods^92^ and lately, the quality of machine learning potentials.^93,94^ Therefore, generating more reference (“training”) data is essential for the development and validation of novel force field parameters. The ever-increasing computational power, including promising advances in quantum computing,^95^ is anticipated to become increasingly important in producing such data, e.g., through accelerated and AI-enhanced first-principles calculations.

However, the reference data must always be carefully evaluated. As simulations scale up in size and complexity, they naturally become more prone to errors arising from either human oversights or previously unexplored regions of conformational space. Consequently, ensuring the transferability and transparency of simulation data is crucial despite technical hurdles. Overall, simulations can be classified into three categories based on their complexity and shareability: (i) simulations that are straightforward to perform, reproduce, and share; (ii) simulations that are challenging but feasible to perform, reproduce, and share; (iii) simulations that are often impossible for a given researcher to perform or reproduce and are similarly difficult to share. The last category thus demands the main attention in the upcoming years.

The need for transparency is even more important, given that the field of MD simulations has significantly lowered the entry barrier, making them increasingly routine and readily utilized as a “black box”. Tools like CHARMM-GUI^96^ and Martinize^97^ exemplify how simulations can be set up and run within mere minutes or hours, which is a stark and positive change from just a few years ago. Similarly, the accessibility of force field parameters in various simulation packages has been improved. For instance, CHARMM-GUI supports the generation of simulation files for major force fields like CHARMM^98^ and AMBER^99^ compatible with not only “home” MD engines^100,101^ but also open-source codes such as Gromacs,^102^ NAMD,^103^ OpenMM,^104^ or Desmond.^105^ The existence of web-based databases sharing molecular topologies and force field parameters also contributes positively.^106,107^ However, there is a potential downside to this accessibility: the widespread availability of simulations could lead to a decline in their quality. The development of multiple user-friendly and open-source codes (which, undoubtedly, is a great step forward) enables the execution of molecular simulations with little knowledge of the underlying physics and MD algorithms. The incorporation of AI may further simplify employing MD, reducing it to merely instructing an AI tool to execute a simulation without the user needing to select a specific force field, software, or simulation parameters. While this could lead to unbiased AI-driven decision-making, it places considerable “responsibility” on the AI tools, including cross-validation and searching for errors.

Nevertheless, human involvement remains an integral part of the development of AI tools. These tools are defined by the model architecture, the data used for training, the objectives they aim to achieve, evaluation metrics, the process of fine-tuning, and subsequent validation. At every stage, human decisions may play a critical role, simultaneously guiding the direction of AI and introducing a degree of subjective bias into the outcomes it produces. For example, selecting a force field for the MD simulation reflects a personal decision influenced by numerous factors. These considerations, whether intentional or subconscious, may also influence the design of AI tools tasked with identifying “the best” force field. Thus, although AI tools are called to automate processes and analyze data volumes beyond human capability, their development and oversight still fundamentally depend on human expertise and judgment. Recognizing the potential diversity of AI tools developed by various teams, one will be expected to test a broad array of those to ensure the consistency and robustness of the results they yield.

Finally, all of these potential AI-driven advancements will also lead to a substantial increase in data generation, with its own set of challenges and implications. Larger, more complex simulations will produce vast amounts of data, demanding storage and analysis. Thereby, this data surge will place significant pressure on the quality of training data for AI tools of various types.^108^ To mitigate the risks associated with the potential data explosion and facilitate efficient error detection, simulations should be subjected to rigorous cross-validation. Such validation becomes feasible only if the training data are collected in shared vast databases, underscoring the need for a unified approach to data management and transparency.

## Challenges in Data Management

Science, driven by both successes and mistakes, thrives on open communication and data sharing. In the realm of MD simulations, managing and accessing vast data volumes is essential yet challenging.^109^ This becomes increasingly critical when considering the need for well-managed and meticulously verified training data for AI applications. While the format of simulation raw data can be easily standardized, managing large data sets at institutional, national, or global levels is increasingly demanding, both technically and financially.^110−112^ Even with standardized formats, storing simulation files proves more complex than storing files such as *.pdb*, which essentially represent the end result of protein structure determination. Consequently, efficient indexing and data storage, imperative for leveraging years of accumulated research, emerges as a critical and mandatory objective to be achieved well before 2050. The data indexing should not be limited to technical aspects but should also account for scientific context, e.g., by highlighting the connections between data sets that are not related from a digital perspective. Although there has been steady progress in this direction over the past years, monumental efforts are still required for significant advancements.

Typically, currently existing databases focus on specific research or methodological areas,^113−119^ like Protein Data Bank,^113^ Uniprot,^118^ or NMR global databank,^119^ to mention only a few. The existence of such databases was central in developing predictive tools such as AlphaFold^48^ or RoseTTAFold.^120^ The idea of databases dedicated to MD models and simulations has been around since at least 1999,^44,45,121−128^ but progress has been limited due to the constraints of resources among researchers, who typically maintain these databases as a secondary task.

In addition to prominent platforms like GitHub (https://github.com/) or GitLab (https://gitlab.com/), repositories such as Zenodo (https://zenodo.org/), Figshare (https://figshare.com/), Open Science Framework (https://osf.io/), Dryad (https://datadryad.org/stash), or Mendeley Data (https://data.mendeley.com/) are increasingly used to store simulation data and codes. However, these repositories are primarily intended for hosting supplementary materials for peer-reviewed publications, such as simulation input files. Storage of hundreds of gigabytes of simulation trajectories presents a nontrivial challenge for these platforms. For example, Zenodo’s upload limit stands at 50 GB in January 2024, which might be insufficient for the trajectories and simulation data of many publications. Although it is theoretically possible to divide the data across multiple repositories, one must upload dozens of segmented parts when dealing with exceptionally large and complexly fragmented data.

**Figure 3:**
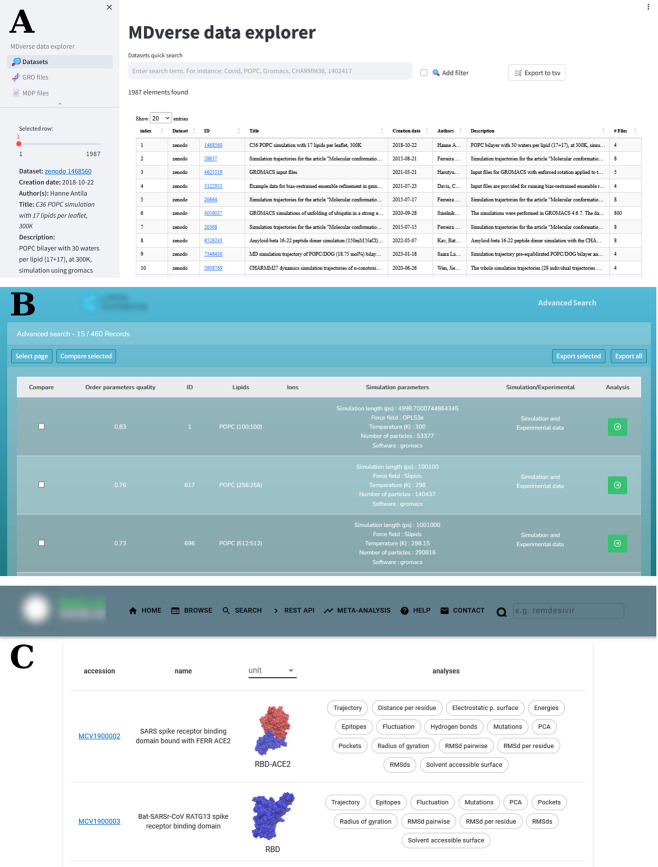
Examples of databases and applications that store and organize molecular dynamics data. (A) MDverse data explorer (https://mdverse.streamlit.app/): an online application navigating through the simulation data collected from Zenodo (https://zenodo.org/), Figshare (https://figshare.com/), and Open Science Framework (https://osf.io/) repositories. (B) NMRlipids databank (https://databank.nmrlipids.fi/): a catalog containing atomistic MD simulations of biologically relevant lipid membranes. The search result for “POPC” is shown. (C) BioExcel-CV19 (https://bioexcel-cv19.bsc.es/): a platform designed to provide web access to atomistic MD trajectories for macromolecules involved in the COVID-19 disease.

Nevertheless, there are already valuable examples that demonstrate how to store and further analyze large amounts of simulation data, Figure 3. Tiemann et al.^129^ recently conducted a systematic analysis of the MD data accumulated in Zenodo, Figshare, and Open Science Framework repositories. They introduced a designated prototype application, https://mdverse.streamlit.app/, that efficiently organizes the collected MD data, Figure 3A. Building on their findings, they offered practical data management tips, such as storing original files uncompressed, providing detailed metadata, and linking to related resources. These recommendations highlight the critical role of well-indexed data; without proper indexing, even accessible data can become useless.^130^

The NMRlipids project,^46,131^https://nmrlipids.blogspot.com/, is, in turn, a promising example of an MD databank, https://databank.nmrlipids.fi/, focused on a well-defined topic. It contains various lipid-membrane simulation data spanning different compositions and modeled conditions, generated using various force fields and MD codes and compared to experiments when available. The interface of this databank is straightforward and intuitive, Figure 3B, suitable for the relatively simple nature of the data. However, more complex data sets would require more flexible and efficient interfaces for effective data navigation. In the biological context, the variability of modeled systems and their composition are apparent, while lipid simulations benefit from the countable and categorizable nature of lipid types. It is thereby more advantageous to offer programmatic access via the API — application programming interface. The NMRlipids project,^46^ alongside other initiatives,^132^ has already made significant progress in this area, allowing a smoother training of related AI models.

Finally, the COVID-19 pandemic showcased how global crises can catalyze the development of centralized databases, as seen with the creation of the pandemic-dedicated database, https://covid.bioexcel.eu/, with related molecular simulations,^132−134^https://bioexcel-cv19.bsc.es/, Figure 3C. This event underscored the value and potential of such databases. Probably, as a consequence, in 2023, a highly ambitious and promising initiative, Molecular Dynamics Data Bank (MDDB, https://mddbr.eu/), received funding, aiming to establish a comprehensive European database for MD data. This initiative seeks to develop an infrastructure for efficient data storage, exchange, analysis, and integration, enhancing the utility and accessibility of MD data.

These positive trends in data storage practices and the rise of larger initiatives are encouraging. However, adhering to FAIR principles^135−137^—ensuring data are findable, accessible, interoperable, and reusable—remains a challenge in practical implementation. Additionally, sharing “failure data”, which could prevent redundant efforts among research groups, is still largely overlooked. Such data are rarely published or made publicly available. To effectively leverage data from centralized databases, it is also essential to establish and implement various controls and “moderators” to oversee and manage these comprehensive repositories. Fortunately, these challenges are mainly well-known, and we possess the necessary knowledge and skills to address them.

## Pathways to 2050

The path of computational physical chemistry toward 2050 relies on several pillars. First, we should rigorously gather, index, organize, and verify the simulation data. Second, an effective search interface is needed to navigate the vast quantities of data, since any database lacking robust search functionality would fall short of its potential. Third, we need to maximize data utilization and foster self-expansion, ensuring that the collected data contribute to advancing the field rather than merely exist as a static digital library. Finally, incorporating control mechanisms into AI-driven research is crucial to avert the accumulation of extensive but flawed data.

Data sharing and accessibility are central to this vision, since transparency is crucial for AI model training and related force field development as it reduces uncertainties in AI applications. The scientific community should be more open with their findings, and publishers should enforce data accessibility. Currently, data availability is often labeled as “available upon reasonable request”, which is not always the case.^138^ The practices of raw data sharing, particularly simulations, have already been implemented by several publishers.^139,140^ More transparent data accessibility should be a standard publishing requirement well earlier than by 2050, of course, taking into account the protection of intellectual property and proprietary data.

Another key aspect is to ensure the accessibility of peer-reviewed articles associated with simulation data to prevent limited accessibility of the necessary data to AI codes. While data sets should have thorough indexing and metadata, related manuscripts often contain vital information not found in the data sets themselves. The current trend toward open-access policies^141^ is expected to result in all scientific journals being open-access by 2050, greatly enhancing data accessibility. Generally, AI should enhance the scientific dialogue and information exchange, e.g., by making scientific tools more widely available, suggesting potential collaborators for specific research projects or transcending possible language barriers.

From another point of view, while AI integration is inevitable, it must be approached with caution. AI can retrieve patterns beyond human perception, but distinguishing real insights from noise is vital. A major concern is a possible amplification of minor AI errors, leading to “false positive” scientific claims. The human bias in AI development could also exacerbate this issue, highlighting the need for skepticism and vigilance. Additionally, scientific data and publications naturally contain errors,^142^ and although they can be found, it is not that simple to quickly fix them.^143,144^ A record number of retracted papers in 2023 is particularly alarming.^145,146^ Thus, AI should be capable of autonomously filtering this information to avoid being misled by it while also being able to discern potential errors from novel insights that lie outside of its training data.

We also should not replace the “knowledge first” concept with the “big amount of data” that AI provides. For instance, while AI algorithms can already streamline the predictions of protein–ligand binding free energies^147,148^ or free energy of permeation through the membrane,^149^ it may struggle with seemingly next-step tasks, such as screening of antimicrobial peptides. Even if we collect all amino-acid sequences known to be effective in a single database and then apply AI to fill in the gaps, the results are far from being guaranteed to be successful. The efficiency of such peptides heavily depends on multiple factors, many of them counter-acting each other, e.g., mechanism of action, selectivity, peptide’s structure, net charge, and amphiphilicity, target microorganism, minimum inhibitory concentration, or possible side effects.^150,151^ Even if AI successfully predicts certain sequences, more is needed to elucidate their functional mechanisms and operational conditions. Thus, a deep understanding of the underlying processes remains more important than blind screening through vast pools of possible combinations (however, it is fair to recognize that if a drug developed by AI proves effective, it represents a huge achievement regardless).

In other words, AI principles should serve to augment and enhance physics-based simulations rather than to replace them. Although AI algorithms can be used to extrapolate the system’s behavior using potentials of arbitrary form, the breakdown and analysis of contributing physical forces, e.g., electrostatics or dispersion interactions, remains crucial. Furthermore, while AI-generated force fields and neural network (“’black box”) potentials show promise,^21^ their application in large-scale simulations needs further exploration. The active development of “black-box” potentials logically necessitates the creation of AI tools that moderate the products of other AI codes.^152^

Attention should also be paid to various biological subtopics. For instance, while proteins and lipids have received significant focus, carbohydrate research is gaining momentum only now^153−156^ (including the development of designated databases^157^), partly due to the important role of glycans in the conformational dynamics and shielding of COVID-19.^158^ The adequately distributed efforts across various fields can foster interdisciplinary communication and facilitate unraveling fundamental scientific questions from multiple perspectives.

In conclusion, as we approach 2050, the integration of AI in computational physical chemistry and biophysics holds tremendous potential for revolutionizing molecular dynamics simulations and data analysis. However, achieving the necessary progress mandates a responsible approach to data management, ensuring a future foundation built on accuracy and integrity. The future computational physical chemist will need to proficiently develop and manage diverse AI tools, addressing various objectives while being mindful of the inherent human biases in AI creation. The potential emergence of artificial general intelligence (AGI)^159^ could further transform not only physical chemistry but also the broader scientific landscape and society. However, the actual definition of AGI and its practical implementation and contribution to physical chemistry remain a topic for future discussions.

## Final Remarks

Molecular dynamics simulations are in constant evolution, and making predictions about their future is an exciting exercise. It is becoming clear that artificial intelligence will significantly influence computational physical chemistry among other fields. By 2050, computational scientists will likely be armed with an array of AI tools. These tools will enable them to navigate through existing data, develop new models, refine current ones, initiate simulations, or even delegate these tasks to AI, including creatively analyzing the outcomes. A critical skill will likely be the ability to effectively communicate with or “prompt” future AI systems. Sharping this skill will presumably require specialized training, including educational programs designed for students. The development of AI tools would also benefit from and perhaps even require robust databases of meticulously accumulated and organized knowledge. These databases provide the indispensable “raw” material for training diverse AI applications and searching intriguing scientific questions emerging from inconsistencies often present in these data.

On a final note, the future is inherently unpredictable. While the emergence of tools like AlphaFold and ChatGPT could be anticipated, their actual impact has been staggering, reshaping not only science but also everyday life. Thus, while our forecasts for future developments might seem reasonable at the moment, new discoveries often lead to even more rapid and unexpected advancements. This accelerated evolution demands from scientists both creativity and adaptability. One such creative approach might be to ask AI itself regarding its vision for 2050. ChatGPT-4’s response to the question “Answer in one sentence how will molecular dynamics and computational physical chemistry look like in 2050” was the following (https://chat.openai.com/share/6025fea1-edc9-493c-8f9c-8a8908f343b2): “By 2050, molecular dynamics and computational physical chemistry will likely be highly advanced, utilizing quantum computing and AI to simulate complex systems with unprecedented speed and accuracy.” Therefore, we can approach the future with optimism, unless ChatGPT-4 was hallucinating.
